# M5C-Related lncRNA Predicts Lung Adenocarcinoma and Tumor Microenvironment Remodeling: Computational Biology and Basic Science

**DOI:** 10.3389/fcell.2022.885568

**Published:** 2022-05-03

**Authors:** Ming Bai, Chen Sun

**Affiliations:** ^1^ Department of Medical Oncology, The First Hospital of China Medical University, Shenyang, China; ^2^ Department of Radiology, Shengjing Hospital of China Medical University, Shenyang, China

**Keywords:** TCGA-LUAD, M5C methylation, long non-coding RNA, tumor microenvironment remodeling, cell migration

## Abstract

**Purpose:** Epigenetic RNA modification regulates gene expression post-transcriptionally. The aim of this study was to construct a prognostic risk model for lung adenocarcinoma (LUAD) using long non-coding RNAs (lncRNAs) related to m5C RNA methylation.

**Method:** The lncRNAs regulated by m5C methyltransferase were identified in TCGA-LUAD dataset using Pearson correlation analysis (coefficient > 0.4), and clustered using non-negative matrix decomposition. The co-expressing gene modules were identified by WGCNA and functionally annotated. The prognostically relevant lncRNAs were screened by LASSO regression and a risk model was constructed. LINC00628 was silenced in the NCI-H460 and NCI-H1299 cell lines using siRNA constructs, and migration and invasion were assessed by the Transwell and wound healing assays respectively.

**Results:** We identified 185 m5C methyltransferase-related lncRNAs in LUAD, of which 16 were significantly associated with overall survival. The lncRNAs were grouped into two clusters on the basis of m5C pattern, and were associated with significant differences in overall and disease-free survival. GSVA revealed a close relationship among m5C score, ribosomes, endolysosomes and lymphocyte migration. Using LASSO regression, we constructed a prognostic signature consisting of LINC00628, LINC02147, and MIR34AHG. The m5C-lncRNA signature score was closely related to overall survival, and the accuracy of the predictive model was verified by the receiver operating characteristic curve and decision curve analysis. Knocking down LINC00628 in NCI-H460 and NCI-H1299 cells significantly reduced their migration and invasion compared to that of control cells.

**Conclusion:** We constructed a prognostic risk model of LUAD using three lncRNAs regulated by m5C methyltransferase, which has potential clinical value.

## Introduction

Each year, 1.8 million people are diagnosed with lung cancer worldwide and about 1.6 million eventually die, indicating a very high mortality rate. Lung cancer is associated with the highest morbidity and mortality rates in China as well, with more than 800,000 new cases every year (Bray et al., 2018). Despite advances in radical surgery, radiotherapy, chemotherapy, targeted therapy, immunotherapy etc., local recurrence and distant metastasis still cannot be achieved (Bray et al., 2018). Lung adenocarcinoma (LUAD) and lung squamous cell carcinoma are the most common types of non-small cell lung carcinoma (NSCLC), of which LUAD accounts for about 70% of all NSCLC cases (Siegel et al., 2020), and is associated with high mortality and recurrence rates (Li et al., 2014). Given the limited understanding of the pathophysiology of LUAD, there is a paucity of effective prognostic indicators (Müller et al., 2016). Although therapies targeting EGFR, TP53, AKT1, KRAS, and PTEN, which frequently undergo mutations and copy number changes in LUAD, have been widely applied in patients with advanced lung cancer (Brose et al., 2002; Bean et al., 2007; Bleeker et al., 2008; Jin et al., 2010), their clinical potential is still limited (Murayama et al., 2016; Schneider et al., 2016). Therefore, there is an urgent need to identify more effective therapeutic targets in order to improve patient outcomes. At present, with the development of high-throughput sequencing and next-generation sequencing, we can get the human genome by gene chip 19000 protein-coding genes of somatic mutation data and copy number amplification; this gives us a more comprehensive understanding of the pathogenesis of lung adenocarcinoma, development disease-related biomarkers provide technical support (Bejjani and Shaffer, 2008).

Epigenetic modifications, including DNA and RNA methylation, genomic imprinting, gene silencing and non-coding RNA activities, regulate gene expression at the transcriptional level (Kaliman, 2019), and are thus involved in multiple pathological processes, including tumorigenesis (Kanwal et al., 2015). Studies show that N6-methyladenine (m6A) and 5-methylcytosine (m5C) RNA methylation play crucial roles in tumor development and progression (He et al., 2019; Ma et al., 2019). High-throughput sequencing has revealed that RNA m5C methylation can modify the sequences of both coding and non-coding RNAs (Chellamuthu and Gray, 2020). The methyltransferase complex that catalyzes DNA/RNA methylation consists of a methyltransferase (“writer”), a methylase demethylase (“eraser”), and an m5C binding protein (“reader ”) (Nombela et al., 2021). There is evidence that the expression levels of m5C-related genes are correlated to the prognosis of lung and pancreatic cancers, indicating that m5C methylation influences tumor growth (Pan et al., 2021; Yu et al., 2021).

Long non-coding RNAs (lncRNAs) are more than 200 nucleotides in length (Qian et al., 2019), and are involved in epigenetic processes such as gene silencing, histone processing, transcriptional regulation and transcriptional interference. Several lncRNAs have been identified in recent years that are involved tumor formation and progression (Bridges et al., 2021). In addition, methylation-related genes affect tumor cell proliferation by regulating the methylation level of specific lncRNAs. For instance, the methyltransferase METTL14 promotes breast tumor development by regulating LINC00942 and its downstream targets (Sun et al., 2020). Nevertheless, little is known regarding the correlation between non-coding RNAs and m5C methylation in LUAD.

In the present study, we used computational biology to identify the lncRNAs regulated by m5C methyltransferase in LUAD, and analyzed the biological functions and pathways associated with the prognostically relevant lncRNAs.

## Methods

### Data Collection

The clinical and transcriptomic data of 576 LUAD patients were obtained from TCGA (https://cancergenome.nih.gov/). The clinical data included gender, survival status, survival time, tumor stage and TNM stage.

### Negative Matrix Factorization Clustering of m5C-lncRNA Gene Set

Thirteen m5C-related genes encoding for lncRNAs were retrieved from literature mining, including NOP2, NSUN2, NSUN3, NSUN4, NSUN5, NSUN7, TRDMT1, TET1, TET2, TET3, ALKBH1, YBX1, and ALYREF (Archer et al., 2016; Blanco et al., 2016; Müller et al., 2016; Cheng et al., 2018; García et al., 2018; Li et al., 2018; Chen et al., 2019; Gao et al., 2019; Janin et al., 2019; Carella et al., 2020; Mei et al., 2020; Sato et al., 2020). After excluding those with median absolute difference <0.5, the correlation of the remaining candidate genes with overall survival was analyzed by the Cox regression model using the “survival” package. The genes with an absolute median >0.5 and *p* < 0.05 were used for NMF dimensionality reduction using the “NMF” package in R (Gaujoux and Seoighe, 2010).

### Weighted Gene Co-expression Network Analysis

A weighted co-expression network was constructed using the WGCNA package in R (Langfelder and Horvath, 2008). PickSoftThreshold was applied to calculate the optimal value of the adjacent function weighting parameter, which was then used as a soft threshold for subsequent network construction. Following construction of a weighted adjacency matrix, the modules of related genes were identified based on hierarchical clustering of the dissimilarity measure (1-TOM) of the topological overlap matrix (Ravasz et al., 2002). The significance of the modules and the mean gene significance within each module were calculated. Finally, the correlation between the co-expression modules and the expression patterns of the resulting subtypes of NMF clustering were calculated.

### Functional Enrichment

The co-expressed module genes were functionally annotated by the GO enrichment analysis and KEGG signaling pathway analysis using the “cluster profile” (Wu et al., 2021).

### Construction of m5C lncRNA Risk Model

The genes significantly associated with the overall survival of LUAD patients were identified by univariate Cox regression analysis (*p* < 0.05) using the LASSO regression algorithm and a risk score model was constructed (Tibshirani, 1997).

### GSVA

GSVA was used to assess the correlation of different gene set scores with m5C methyltransferase-related scores.

### Immune Infiltration Analysis

The relative proportion of immune-infiltrating cells in the two risk groups was analyzed using the CIBERSORT (Newman et al., 2019; Steen et al., 2020), EPIC (Racle et al., 2017), quanTIseq (Finotello et al., 2019), MCPcounter (Becht et al., 2016), XCELL (Aran et al., 2017), and 和TIMER (Li et al., 2016) algorithms. The differences in immune responses were visualized through heat maps.

### Cell Culture

NCI-H460 and NCI-H1299 cell lines were provided by the Shanghai Cell Bank of the Chinese Academy of Sciences. The cells were cultured in DMEM supplemented with 10% fetal bovine serum at 37°C in a 5% CO_2_ incubator. The cells were seeded in a 6-well culture plate at the density of 4 × 10^5^ cells per well and cultured overnight for subsequent experiments.

### Cell Transduction

NCI-H460 and NCI-H1299 cells in the logarithmic growth phase were harvested, seeded in 6-well plates, and transduced with lentiviruses expressing si-NC, si1-LINC00628 or si2-LINC00628 at the multiplicity of infection of 10. After 24 h of culture, 2 μL polybrene was added to a final concentration of 5 μg/ml for 1–2 weeks, and the medium was changed every 8–12 h. The stably transduced cells expressing GFP were detected 72–96 later under a fluorescence microscope, and expanded further.

### RT-PCR

LINC00628 silencing in the transduced cells was analyzed by RT-PCR using the following primers: Forward—5′-CAGTGGGGAACTCTGACTCG-3′ and Reverse—5′-GTGCCTGGTGCTCTCTTACC-3′.

### Wound Healing and Transwell Assays

The *in vitro* migration of the control and LINC00628-knockdown cell lines were determined by the wound healing and Transwell assays respectively.

## Results

### Identification of M5C-Related lncRNA Molecular Subtypes in LUAD Based on NMF Classification

**FIGURE 1:**
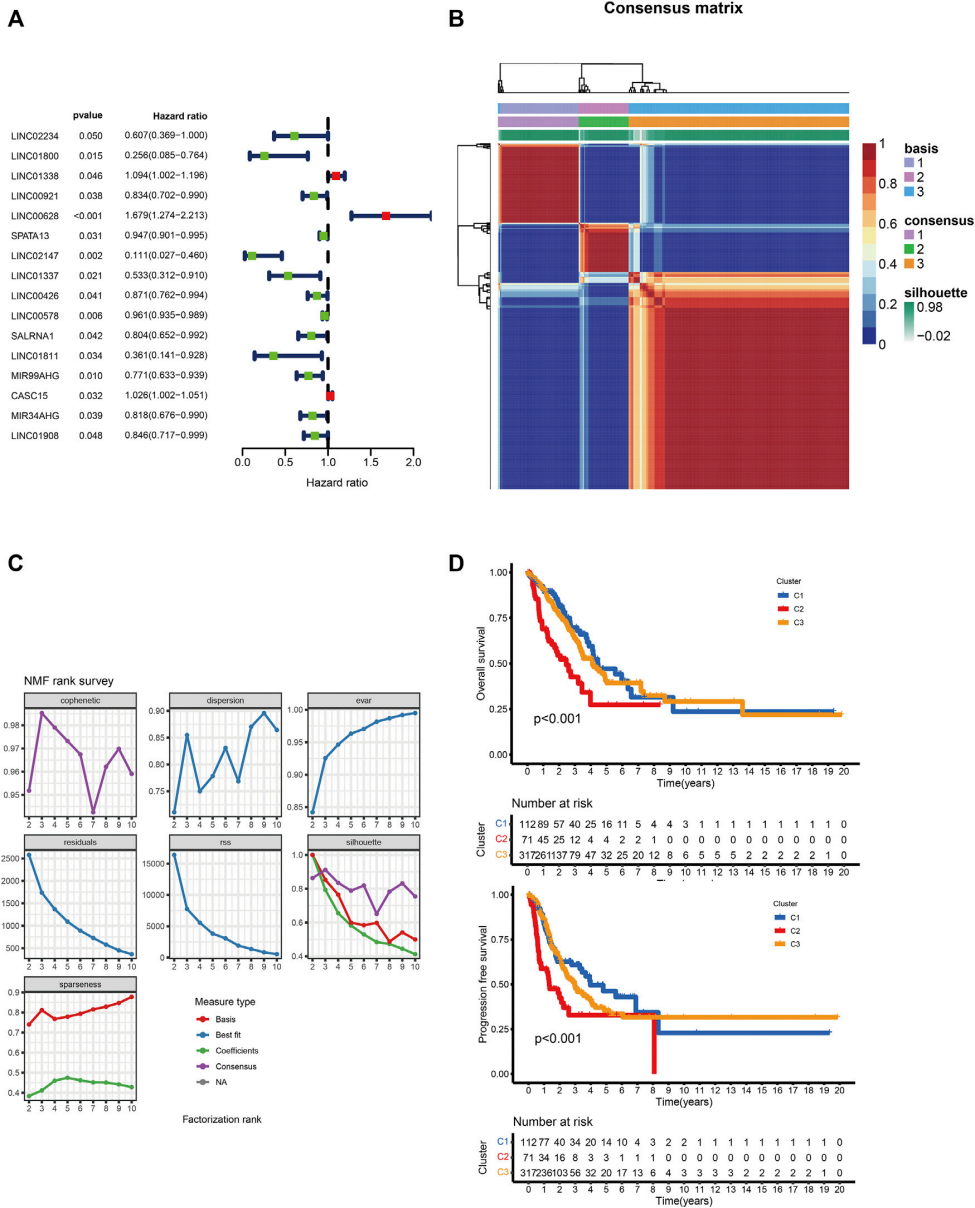
**(A)** Univariate Cox regression analysis of 16 lncRNAs and overall survival. **(B)** Consensus map of NMF clustering. **(C)**Consensus clustering parameters. **(D)** Overall and disease-free survival of C1, C2 and C3 clusters.

We identified 185 M5C-related lncRNAs with Pearson correlation coefficients greater than 0.4 (Supplementary Table S1), of which 16 were significantly associated with LUAD prognosis (*p* < 0.05; Figure 1A). NMF clustering was then performed on these lncRNA-related genes with 50 iterations, which identified nine clusters. The number of collections (k) was 2–10, and the minimum sample of each group was set to 10. According to cophenetic, dispersion and silhouette, we selected the ideal clustering group as 3 (Figures 1B,C). The grouping details are shown in Supplementary Table S2. The molecular subtypes of lncRNAs based on m5C methylation patterns was associated with significant differences in overall and disease-free survival (Figure 1D; log-rank *p* < 0.05).

### Biological Characteristics of the lncRNA-Related Genes

**FIGURE 2:**
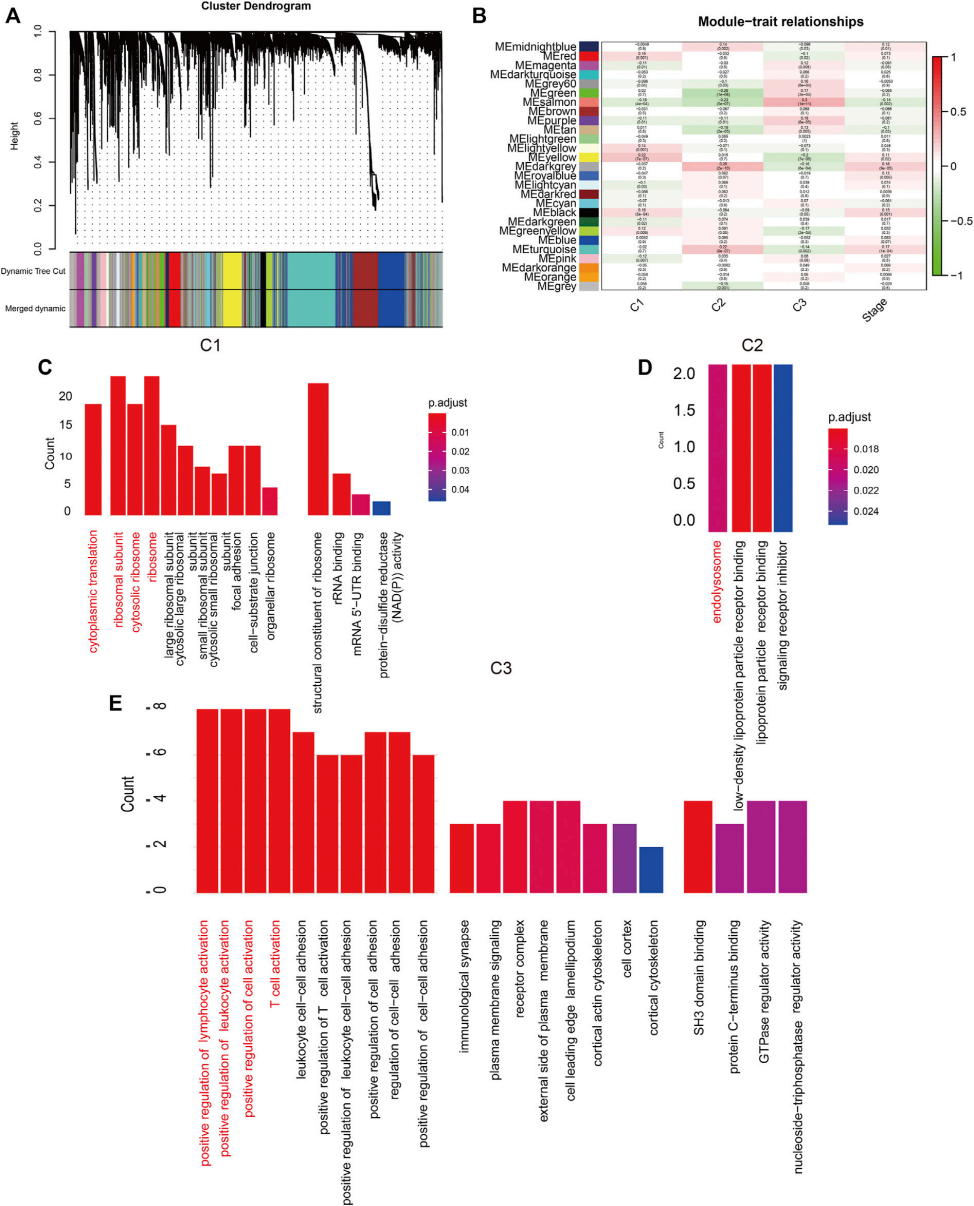
**(A)** Hierarchical clustering tree. Each leaf represents a gene and each branch represents a co-expression module. **(B)** Twenty-seven co-expression modules were included. **(C–E)**: Significantly enriched biological processes, molecular functions and cellular components in C1, C2, and C3 groups.

**FIGURE 3:**
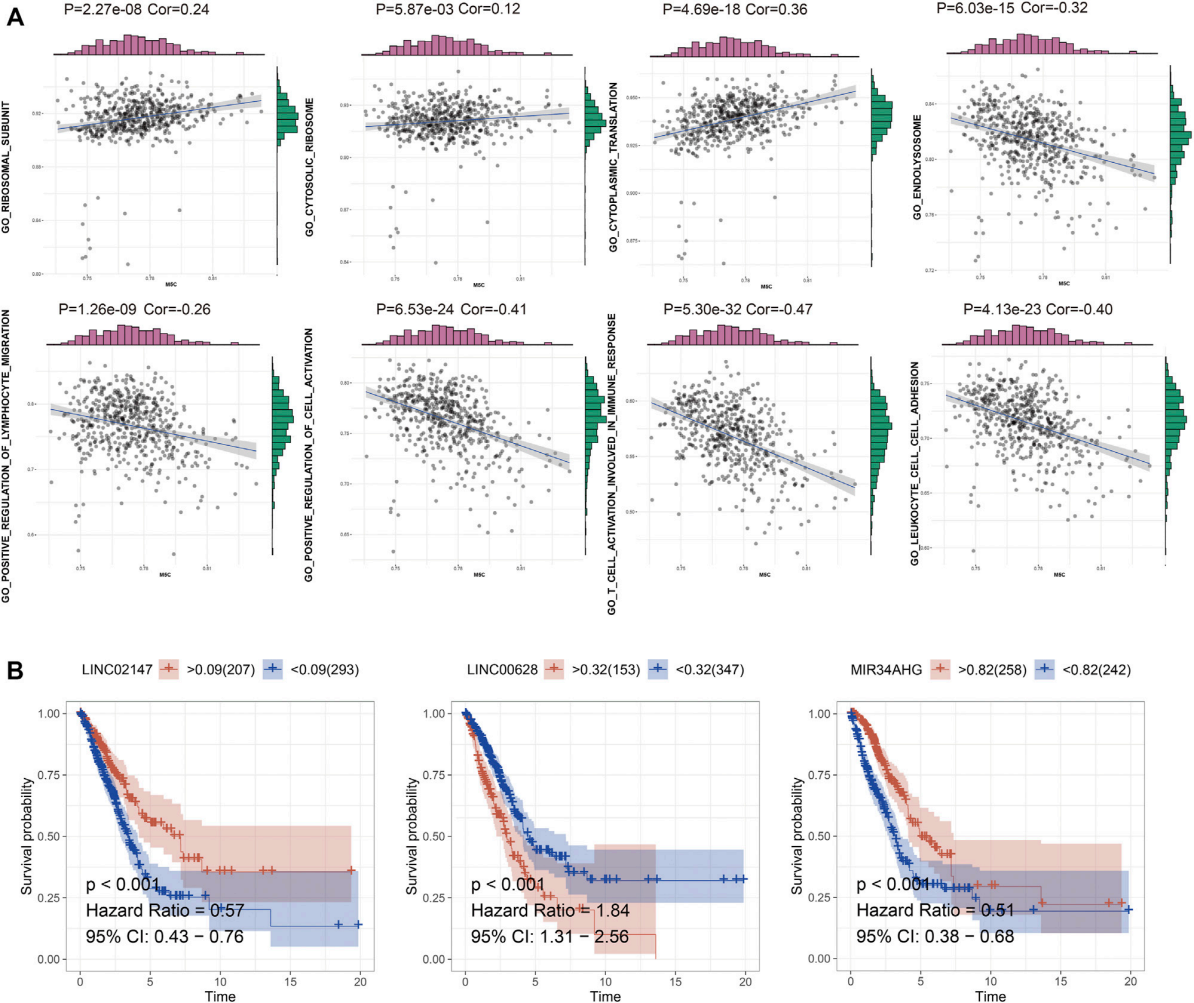
**(A)** GSVA of m5C RNA methylation dataset and enriched functions in C1, C2, and C3. **(B)** The survival curve of the LINC02147, LINC00628, and MIR34AHG.

WCGNA of the lncRNA-related genes revealed 27 co-expression modules with *β* value set to 3, and genes in the smallest module set to 30 (Figure 2A). The C1 cluster had the strongest correlation with the yellow module (Figure 2B; Cor = 0.2). Functional annotation of the top 20 genes most closely related to C1 in the yellow module indicated that these genes are associated with ribosomal subunits (Figure 2C). The C2 cluster was strongly correlated with the black-gray module (Figure 2B; Cor = 0.28), and the top 20 genes were enriched in lysosome-related functions (Figure 2D). The C3 cluster showed significant correlation with the salmon color module (Figure 2B; Cor = 0.3), and was mainly associated with lymphocyte regulation (Figure 2E). To directly demonstrate the relationship between m5C and the biological function of each expression pattern signature, we performed GSVA for each gene set and obtained the scores for m5C methyltransferase. As shown in Figure 3A, m5C RNA methyltransferase, ribosome subunits, lysosomes, and lymphocyte migration were closely associated.

### Construction of a LUAD Predictive Model Based on lncRNA-Related Predictive Genes

**FIGURE 4:**
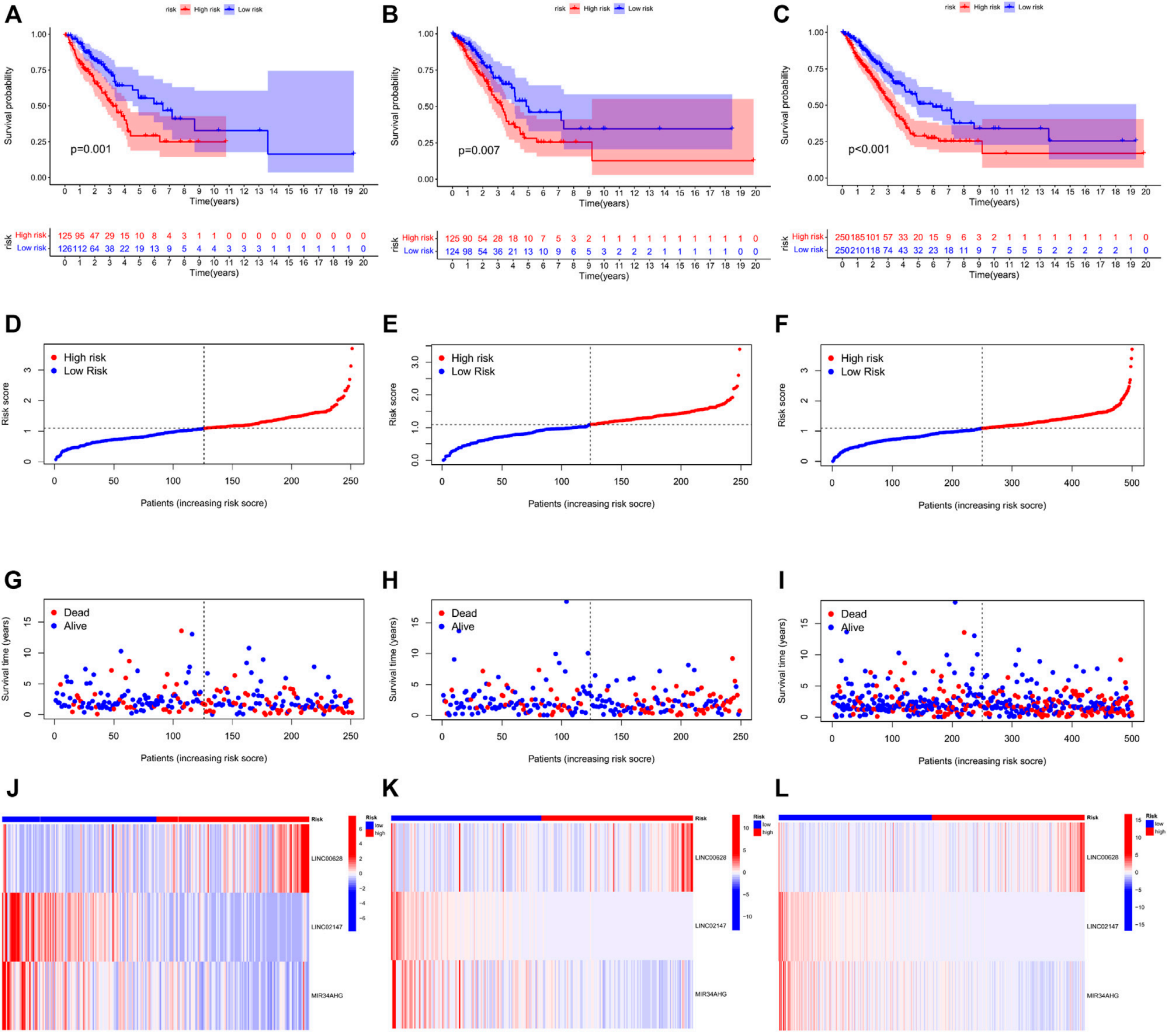
Risk score distribution and survival status in the training, validation and total TCGA-LUAD sets. The survival status, risk score, gene expression level in the risk score and follow-up time of each sample are shown.

TCGA-LUAD cohort was randomly divided into the training and validation sets. Briefly, the data samples were sorted in the ascending order by ID and random numbers were assigned to each sample using SPSS. Both sets were similar in terms of age, clinical-stage, follow-up time and survival, as well as the gene expression profiles (Supplementary Table S3). LASSO regression was used to construct the predictive model by incorporating the 16 prognostic genes and overall survival rates. LINC00628, LINC02147, and MIR34AHG were identified as the independent prognostic factors and used for the final predictive risk model. The risk score for each sample was calculated as 0.45 * exp LINC00628—2.67*expLINC02147—0.30 * exp MIR34AHG. The survival curves for each independent prognostic factor are shown in Figure 3B. The patients in the training and validation sets were classified into the high- and low-risk groups based on the risk scores, which showed significant differences in survival in both sets (Figure 4; *p* < 0.05).

### Correlation of Risk Scores With Immune Checkpoints and Immune Infiltration

**FIGURE 5:**
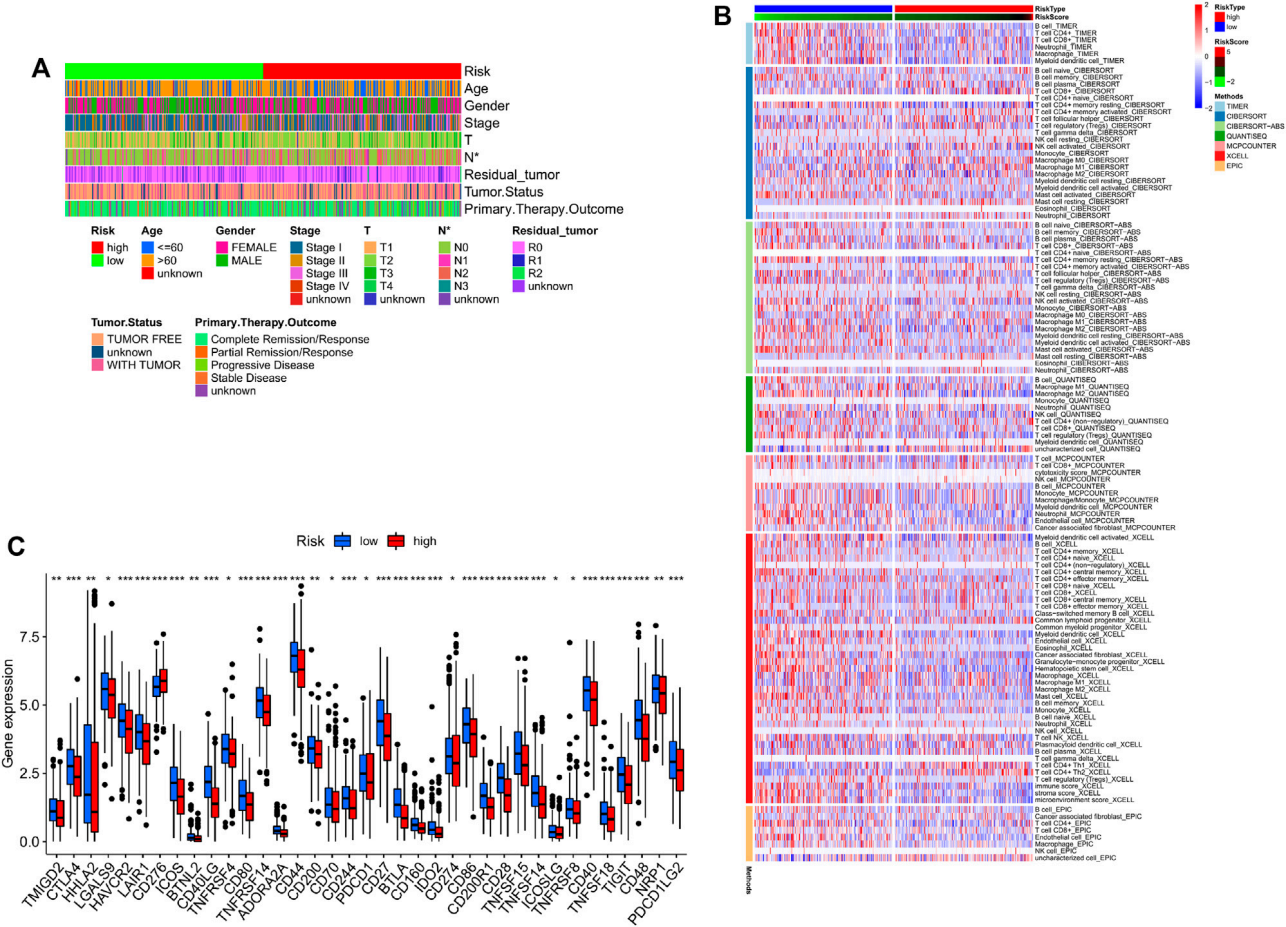
**(A)** Clinical phenotypes of patients in the two risk groups. **(B)** Immune infiltration in the high- and low-risk groups. **(C)** Immune checkpoint expression levels in the two risk score groups.

The LUAD patients were divided into high- and low-risk groups according to the median risk score, and their clinical characteristics are summarized in Figure 5A. Immune cell infiltration was assessed using CIBERSORT, EPIC, quanTIseq, MCPcounter, XCELL and TIMER programs. As shown in the heatmap in Figure 5B, the proportion of infiltrating macrophages was lower in the high-risk group. In addition, the infiltration levels of CD8^+^ T cells and M1/M2 macrophages were also significantly different between the two risk groups, as were the expression levels of immune checkpoints such as CTAL4 and CD276. This finding suggests that risk scores can be used as immunotherapy biomarkers for LUAD (Figure 5C).

### LINC006328 Regulates Migration of LUAD Cells

**FIGURE 6:**
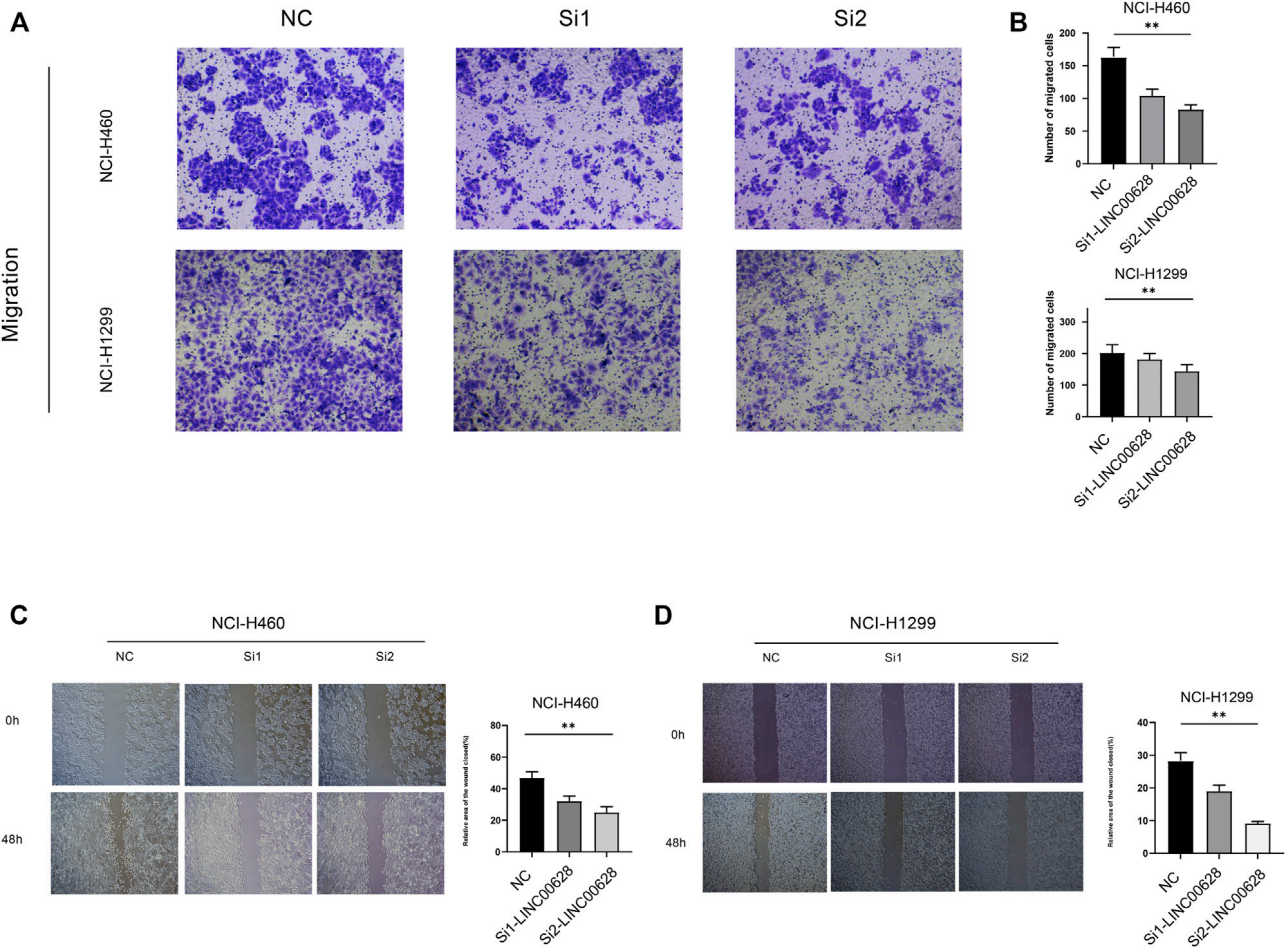
**(A,B)** Transwell assay of si1-LINC00628 and si2- LINC00628 groups. **(C,D)** Wound healing assay of si1-LINC00628 and si2-LINC00628 groups. *p < 0.5, **p < 0.01, ***p < 0.001, ****p < 0.0001, ns, no significance.

To further determine the biological significance of LINC00628 in LUAD, the gene was silenced in the NCI-H460 and NCI-H1299 cell lines using two specific siRNA constructs. The LINC006328-knockdown cells showed significantly reduced invasion (*p* < 0.05; Figures 6A,B) and migration (*p* < 0.05; Figures 6C,D) rates compared to the control groups in the Transwell and wound healing assays respectively. Thus, LINC00628 likely functions as an oncogene in LUAD and promotes tumor cell migration and invasion.

## Discussion

Although surgery, radiotherapy and chemotherapy have prolonged the survival of LUAD patients, the prognosis is far from satisfactory. Early diagnostic markers for LUAD are lacking due to incomplete understanding of its pathological basis. Therefore, it is crucial to explore the genetic and epigenetic factors involved in LUAD in order to identify novel therapeutic targets and diagnostic biomarkers (Sung et al., 2021).

RNA methylation is a critical epigenetic modification involved in post-transcriptional gene regulation, and includes m6A, m1A, m5C, m7G, and other types. Methylation of the fifth cytosine (m5C) is particularly widespread (Traube and Carell, 2017; Mongan et al., 2019; Xie et al., 2020), and participates in various physiological and pathological processes (Li et al., 2017; Zhao et al., 2017; Bohnsack et al., 2019). The m5C modification in tRNA and rRNA regulates translation and the quality of ribosome biosynthesis respectively. In addition, methylation of 5C in mRNA affects its structure, stability and translation.

LncRNAs consist of more than 200 nucleotides and were initially considered “junk sequences” with no specific biological functions. However, recent studies show that lncRNAs are widely expressed in human cells and are associated with tumor development. In fact, several lncRNAs have been identified as potential prognostic markers and therapeutic targets for multiple tumors (Nandwani et al., 2021). In addition to post-transcriptional regulation of protein-coding RNAs, the lncRNAs can also bind to proteins and molecular scaffolds and affect tumor growth through *in situ* regulation and molecular convergence (Kopp and Mendell, 2018; Nandwani et al., 2021). In the present study, we used computational biology to identify lncRNAs that are regulated by m5C methyltransferase, and determined their prognostic relevance in LUAD. Bioinformatics analysis further indicated that the m5C scores of these lncRNAs were correlated with ribosome subunit, cytosolic ribosome, cytoplasmic translation, endolysosome and lymphocyte migration.

We identified 185 lncRNAs related to genes encoding m5C methyltransferase, of which 16 were prognostically relevant, including LINC00628 that was significantly correlated with multiple m5C methyltransferases. Therefore, we hypothesized that LINC00628 is regulated by m5C methylation during LUAD progression. Indeed, knocking down LINC00628 in two LIAD cell lines significantly reduced their migration and invasion rates *in vitro*, which is suggestive of an oncogenic role in LUAD. However, studies show that LINC00628 can function as an oncogene or tumor suppressor in different cancers (Zhang et al., 2016; He et al., 2018). For example, overexpression of LINC00628 inhibited the proliferation and migration of osteosarcoma cells (He et al., 2018), whereas its knockdown in gastric cells had a similar inhibitory effect (Zhang et al., 2016). Consistent with our results, Xu et al. found that LINC00628 promoted LUAD progression by targeting the LAMA3 promoter region (Xu et al., 2019). In addition, we also identified LNC02147 as a protective factor in LUAD.

However, a major limitation of our study is that our findings are based on bioinformatics analysis, and will have to be validated on cross-cohort samples. In addition, the mechanisms underlying the function of m5C-related lncRNAs in LUAD also need to be explored.

## Conclusion

We identified prognostically relevant LUAD-related lncRNAs that are regulated by m5C methyltransferase, and constructed a predictive model based on these lncRNAs. Our findings provide a basis for further research on the role of m5C modification in LUAD.

## Data Availability

Publicly available datasets were analyzed in this study. This data can be found here: The datasets TCGA-LUAD for this study can be found at http://cancergenome.nih.gov/.

## Abbreviations

GSEA, gene set enrichment analysis; LASSO, least absolute shrinkage and selection operator; LUAD, lung adenocarcinoma; m5C, 5-methylcytosine RNA methyltransferases; NMF, non-negative matrix factorization; WGCNA, weighted correlation network analysis.
